# Is radical surgery of an inverted papilloma of the maxillary sinus obsolete? a case report

**DOI:** 10.1186/s13256-016-1114-1

**Published:** 2016-12-01

**Authors:** Vedat Yildirim, Niels Christian Pausch, Dirk Halama, Heinz-Theo Lübbers, Ayhan Yildirim

**Affiliations:** 1 Department of Oral, Craniomaxillofacial and Facial Plastic Surgery, University Hospital of Leipzig, Liebigstr. 12, 04103 Leipzig, Germany; 2 Private Practice for Oral and Maxillofacial Surgery, Archstr. 12, 8400 Winterthur, Switzerland; 3 Clinic for Cranio-Maxillofacial Surgery, University Hospital of Zurich, Frauenklinikstr. 24, 8091 Zurich, Switzerland

**Keywords:** Inverted papilloma, Endoscopic resection, Radical approach, Caldwell–Luc, Case report

## Abstract

**Background:**

Sinonasal inverted papilloma is a locally aggressive tumor arising from the Schneiderian membrane which lines the nasal cavity and paranasal sinuses. Aggressive surgical approaches, such as lateral rhinotomy, were used until recently for complete removal of the inverted papilloma. Currently, endoscopic resection is the gold standard in the treatment of inverted papilloma. However, there are situations that justify an open approach. For example there are studies that report a higher postoperative recurrence rate after endonasal endoscopic resection, particularly in the treatment of recurrent diseases. While endoscopic resection performed by an experienced surgeon is definitely a minimally invasive therapy, an open approach is not necessarily associated with functional and aesthetic disadvantages.

This case report describes the treatment of inverted papilloma by an open approach. This has been described before but the new gold standard of endoscopic resection has to be taken into account before any treatment decision is made nowadays.

**Case presentation:**

Contrast-enhanced magnetic resonance imaging of the head and neck area was indicated in a 72-year-old white German man who presented with suspected squamous cell carcinoma of his lower lip. Magnetic resonance imaging additionally revealed a 3×2 cm^2^ polycyclic arranged mucosal thickening with cystic and solid contrast affine shares at the antral laterocaudal area of his right maxillary sinus, extending from his right lateral nasal wall to his maxillary sinus floor.

He received antral polypectomy with medial maxillectomy via a unilateral LeFort I osteotomy approach. His pterygoid plate was preserved. A histological examination demonstrated a tumor composed of hyperplastic squamous epithelium protruding into the stroma (surface epithelial cells grew downward into the underlying supportive tissue), thus producing a grossly convoluted cerebriform appearance. Two weeks later, the patient regained a well-formed maxilla without any restrictions. He has remained disease-free for 25 months following the surgery and surveillance was continued in our tumor clinic.

**Conclusions:**

Endoscopic resection of an inverted papilloma continues to be the gold standard. However, some cases require a radical approach. This does not necessarily increase patient morbidity.

## Background

Inverted papilloma (IP) is an uncommon lesion that accounts for less than 0.5 to 4 % of all sinonasal tumors [1]. The treatment of choice for IP involves a wide excision using either an open or endoscopic approach. Candidates for endoscopic surgery are appropriately selected patients with a limited disease spread on their ethmoid sinuses, their lateral nasal wall, or their medial maxillary wall. Contraindications to endoscopic resection included massive skull base erosion, intradural or intraorbital extension [2], abundant scar tissue from previous surgery, and/or associated squamous cell carcinoma [3]. Extensive tumors usually require medial maxillectomy via a lateral rhinotomy approach. This case report reassesses the treatment procedure and the outcome based on the case of one patient treated in our clinic.

## Case presentation

A 72-year-old white German man presented with a 5-month history of a growing lip mass. A review of his medical history revealed that he had arterial hypertension, diabetes mellitus, peripheral arterial disease, and a one pack-daily cigarette smoking history. Four years earlier, he underwent a resection for prostate carcinoma.

**Fig. 1:**
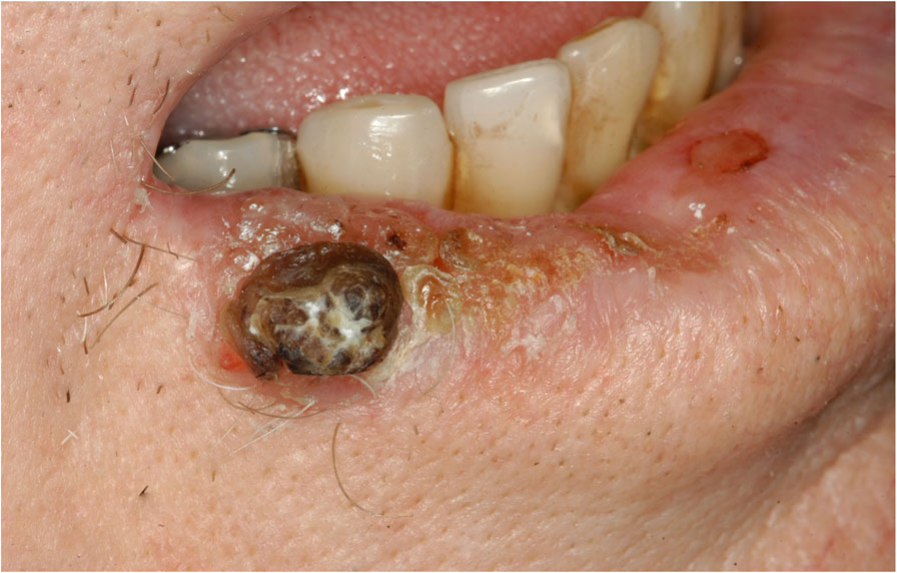
Affection in the right lower lip. This finding initially led the patient to consult a specialist and finally this was the reason inverted papilloma could be diagnosed by magnetic resonance imaging

A clinical examination revealed no abnormalities other than a firm mass on the right side of his lower lip (Fig. 1), without palpable cervical lymphadenopathy. Because squamous cell carcinoma of the lower lip was suspected, he was scheduled for contrast-enhanced magnetic resonance imaging (MRI) of his head and neck area. The MRI showed next to a tumorous contrast-affine process of approximately 1.2 cm in diameter on his right lower lip, a 3×2 cm^2^ polycyclic arranged mucosal thickening with cystic and solid contrast affine shares at the antral laterocaudal area of his right maxillary sinus, extending from his right lateral nasal wall to maxillary sinus floor. There was no evidence of bony erosion or other cervicofacial lesions. Under general anesthesia, he received the following procedures: an excisional biopsy at the lip lesion with 5 mm safety margins and bilateral sentinel lymph node biopsy, and antral polypectomy with medial maxillectomy via a unilateral LeFort I osteotomy approach with preservation of his pterygoid plate. Histological examination of the hematoxylin-eosin specimens demonstrated well-differentiated squamous cell carcinoma of the lip (not shown). The antral lesion was a tumor composed of hyperplastic squamous epithelium protruding into the stroma (surface epithelial cells grew downward into the underlying supportive tissue), thus producing a grossly convoluted cerebriform appearance.

He was hospitalized for 2 weeks (12 days) after the surgery and did well postoperatively. Two weeks later, he regained a well-formed maxilla without any restrictions. Eleven months after our therapy, a cutaneous malignant melanoma on his right upper arm was diagnosed (Breslow index 1.9 mm, Clark level IV) and he underwent a tumor resection and axillary sentinel lymph node biopsy in the clinic for dermatology of the University of Leipzig. He has remained disease-free for 25 months following the first surgery and surveillance was continued in our tumor clinic. This case substantiates our procedure of radical resection.

Our patient presented with an asymptomatic IP of his maxillary sinus accidentally found with squamous cell carcinoma of his lower lip. This case required particular attention because a carcinoma could have been associated with the IP. A relatively aggressive approach for IP was, therefore, applied at the same time as the cancer surgery.

## Discussion

Currently, no generally accepted treatment for IP of the sinonasal area exists. Some authors prefer an endonasal micro-endoscopic removal [4], while others prefer a radical resection (Caldwell–Luc) that is similar to our treatment [5]. The decision depends on the position, dimensions, stage, and recurrence of the tumor. Some authors believe that radical therapy of the IP in this area causes functional and aesthetic problems for the patient. Since very little data exist concerning the treatment and outcome for different treatments of IP in the sinonasal area, it is difficult to find the best solution for each patient. Because of the position of the tumor, radical resection was preferred in this case. Postoperative readjustment of the prosthesis was without functional constrictions. Our case shows that radical resection of tumors in this location does not necessarily imply serious functional and aesthetic impairment for the patient. It is notable that the reported relationship between IP and squamous cell carcinoma was observed in this case [6].

It is very important to correctly diagnose a tumor in its early stage to cure or treat the patient as soon as possible. A wrong diagnosis for IP has fatal consequences for patients. It must be decided from case to case which treatment is the best one for the patient. If a tumor is detected at an early stage and in an accessible location, endonasal micro-endoscopic surgery is a very elegant solution and can be done by any accordingly trained surgeon, mostly an otorhinolaryngologist. To achieve low recurrence rates of IP (and oncocytic papilloma), surgeons performing an endoscopic approach should consider drilling, cauterizing, or completely excising the bone underlying the tumor base instead of mucosal stripping alone [7]. Whenever the location or stage of the tumor prohibits the endonasal technique, radical resection is the best option and it does not necessarily cause significant functional and aesthetic problems for the patient.

Currently, the treatment for IP is surgical but the type of surgical approach is still controversial. Endoscopic surgery has become the gold standard for the treatment of the vast majority of IP. Antral polypectomy with medial maxillectomy via a unilateral LeFort I osteotomy is much more aggressive than the other approaches but might be justified especially in higher stages of disease [8]. There is a likely possibility of cutting through the tumor’s stalk and the oscillatory saw dispersing the tumor cells around the tumor site, which leads to a higher risk of recurrence. However, the risk of dispersal of tumor cells during surgery is also present during the other approaches. A well-done MRI and an expert surgeon can minimize these risks.

Another discussion is about the possible malignant transformation of IP. Some authors also recommend radiotherapy for advanced and/or recurrent papillomas [9]. This of course can be performed in addition to surgery or even as the only treatment.

**Fig. 2:**
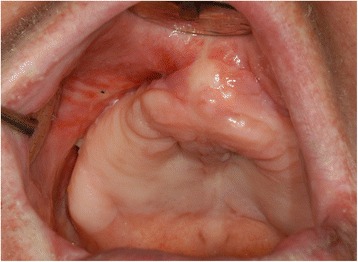
Situation of the maxilla 14 days after the end of treatment. No relevant disturbance to the patient’s daily life occurred as a result of the treatment

More studies have to be performed in order to analyze and record the recurrence rates and complications for different approaches [1, 8]. Today we recommend consideration of our approach in patients with advanced disease and/or a high risk for other tumors. As shown, it is not necessarily associated with any significant permanent restriction for the patient (Fig. 2).

In the light of the above-mentioned options and the different specialties possibly involved we strongly recommend treating IP within a multidisciplinary tumor board setting. Any treatment choice should only be made under consideration of the above-mentioned options of surgery (either endoscopic or open) and/or radiotherapy. In particular, an endoscopic surgeon needs to be “on board” since whatever treatment choice is made the endoscopic technique is always to be considered for at least preoperative biopsy and follow-up [1].

## Conclusions

Endoscopic resection of an IP continues to be the gold standard. However, some cases require a more radical approach. This does not necessarily increase patient morbidity. Even so, endoscopic resection offers a great new treatment option; radical surgery is definitely not obsolete. Taking into account that radiotherapy is also worthy of discussion, a multidisciplinary discussion of any case is strongly recommended.
